# Arterial switch with pulmonary artery banding: A staged repair strategy for transposition of great arteries with complex intracardiac anatomy

**DOI:** 10.1016/j.xjon.2026.101622

**Published:** 2026-02-10

**Authors:** Nathanael Shraer, Sergii Galych, Aubin Nassif, Ségolène Bernheim, Alexander Moiroux-Sahraoui, Neil Derridj, Damien Bonnet, Olivier Raisky

**Affiliations:** a Department of Pediatric Cardiac Surgery, Necker Sick Children Hospital, Paris, France; b Department of Pediatric Cardiology, Necker Sick Children Hospital, Paris, France

**Keywords:** congenital heart surgery, transposition of great arteries, ventricular septal defect

## Abstract

**Objective:**

We present initial results of arterial switch operation (ASO) with pulmonary artery banding (PAB), followed by intracardiac repair for patients with transposition of great arteries (TGA) associated with unusually complex intracardiac anatomy.

**Methods:**

Between 2000 and 2023, these patients (1.9% of 1744 TGA in our institution) were retrospectively reviewed. Staged repair was either strategy A (2000-2018; 1. PAB, 2. ASO + intracardiac repair) or strategy B (2014-2023; 1. ASO + PAB, 2. Intracardiac repair); aortic arch repair could be associated. We compared those strategies.

**Results:**

Thirty-five patients were included (18 in strategy A, 17 in strategy B). After first stage, unplanned reoperation incidence was greater with strategy A (n = 6, 27.8%; 95% CI, 7.1-48.5) than B (n = 1, 7.3%; 95% CI, 0.0-21.1, *P* = .043). Patients who received strategy A underwent second-stage repair earlier (time interval: 8.1; interquartile range [IQR], 2.9, 9.4 vs 15.3 months; IQR, 12.1, 18.2; *P* = .006) with poorer weight (6.3; IQR, 4.4, 7.7 vs 9.9 kg; IQR, 8.5, 10.7; *P* = .005) and deeper cyanosis (75.0%; IQR, 70.5, 76.5 vs 87.0%; IQR, 85.25, 89.0, *P* = .005). They had longer stays in the intensive care unit (7.5 days; IQR, 5.75, 9.0 vs 2.0 days; IQR, 2.0, 2.75; *P* = .002), mechanical ventilation (2.5; IQR, 1.0, 6.0]) versus 1.0 day; IQR, 0.0, 1.0; *P* = .006), and inotrope duration (4.0; IQR, 2.0, 6.0 vs 1.0 day; IQR, 0.25, 1.0), *P* = .002) and more ventilatory complications (55.6% [n = 10] vs 17.6% [n = 3], *P* = .049). Death from cardiac cause occurred in 1 patient in each group (*P* = 1).

**Conclusions:**

Early ASO + PAB procedure, over PAB only, for patients with TGA with complex intracardiac anatomy leads to early anatomical repair with greater oximetry, less unplanned reoperation, and easier short-term outcomes.

**Figure:**
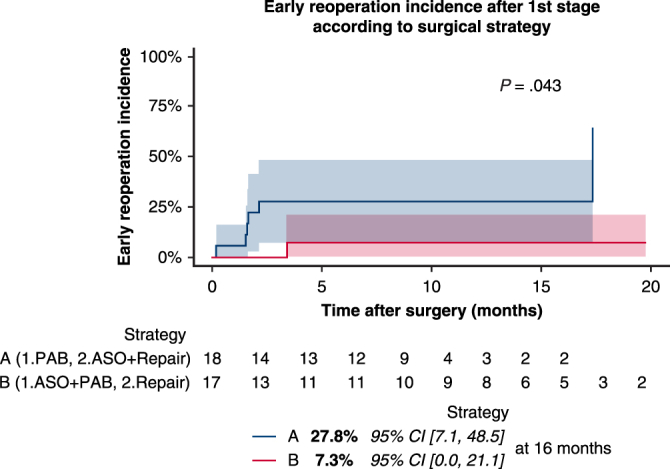
After 1st stage, early reoperation was 27.8% (strategy A) vs 7.3% (strategy B) (*P* = .043).

**Central Message**

Performing ASO with PAB allows early anatomical repair with greater oximetry, prevents unplanned reoperation, and leads to shorter ICU stays with less ventilatory complications.

**Perspective**

Patients with TGA associated with unusually complex intracardiac anatomy are difficult to manage. Staged surgery with early ASO + PAB, versus PAB only, seems to allows greater oximetry until patient's sufficient growth for a safe and uneventful intracardiac repair.

Management of transposition of the great arteries (TGA) associated with ventricular septal defect (VSD) can still be a matter of debate.^1^, ^2^, ^3^, ^4^ Various factors may influence the choice between a 1-staged repair (arterial switch operation [ASO] with VSD closure) or a classical 2-staged repair (1/pulmonary artery banding [PAB], 2/ASO with VSD closure). Among them, intracardiac anatomy, aortic arch anatomy, and center experience all play a role in this decision. However, it has been reported that whenever the situation seems appropriate, single-stage should be preferred, because it significantly decreases the overall rate of morbidity.^5^, ^6^, ^7^, ^8^, ^9^ Indeed, the 2-stage approach presents the risk of 2 surgeries, the PAB damaging the neoaortic valve, and it has been reported that PAB is associated with increased risk of early death in this population of patients.^10^ Moreover, there is now strong evidence that late dilatation and regurgitation of the neoaortic root and valve occur more in patients with a history of PAB.^11^, ^12^, ^13^, ^14^, ^15^, ^16^, ^17^

However, even in high-volume centers, there are some cases in which the single-stage approach in neonates seems too complex and/or risky if performed too early (ie, risk of altering ventricular architecture and causing dysfunction, risk of injuring the atrioventricular bundle, uncertainty of biventricular repair^1^^,^^3^), In those peculiar situations, a 2-stage repair is generally preferred but remains with the aforementioned inherent risks.

We propose a “modified” 2-staged approach, with the first surgery comprising ASO with PAB and the second surgery the intracardiac repair with VSD closure and pulmonary artery (PA) debanding. We present our early experience with this novel 2-staged approach in our institution (1/ASO + PAB, 2/VSD closure and PA debanding), and compare it with our previous classical strategy (1/PAB, 2/ASO, VSD closure and PA debanding).

## Methods

### Patients

Between February 2000 and June 2023, 1744 patients were diagnosed with TGA in our institution. Among them, 540 had TGA associated with VSD (31.0%), in whom 171 had concomitant aortic arch anomaly (9.8%). These 540 patients benefited of a single-stage repair, except for 35 patients defined as TGA with unusually complex intracardiac anatomy (6.3% of TGA with VSD; 1.9% of all TGA) who were retrospectively reviewed. This included complex VSD (multiple with apical Swiss cheese, extremely large inlet or outlet), double-outlet right ventricle with unusual expected difficult channeling, atrioventricular valve straddling and overriding or small right ventricle (RV) (defined by a tricuspid valve [TV] annulus z score ≤−3) with uncertainty of biventricular repair. Patients could have associations among these defects. Aortic arch anomaly could also be present.

Our initial institutional strategy (first stage: PAB [±aortic arch repair], second sage: ASO and intracardiac repair) was performed between 2000 and 2018 on 18 patients (strategy A). These served as a reference for comparison with our novel strategy (first stage: ASO + PAB [±aortic arch repair], second stage: intracardiac repair) (strategy B, n = 17).

All patients were operated at Necker Sick Children Hospital, and parental consent was waived from each patient. The study was approved by the local ethics committee at Necker Sick Children Hospital, institutional review board number 20240705143339, approved July 5, 2024.

### Surgical Technique

#### First stage

##### Strategy A

PAB was performed through sternotomy or through left thoracotomy when associated with aortic coarctation repair. A 1- to 2-mm width Gore-Tex band was passed around the PA trunk and tied around it away from the valve and the bifurcation, and banding was applied according to the Trusler formula^18^ (circumference of 20 mm + 1 mm/kg).

When the aortic arch was extensively hypoplastic, it was enlarged with treated autologous pericardial patch through sternotomy on cardiopulmonary bypass with moderate hypothermia (24 °C) and innominate artery continuous perfusion. PAB was established with the same rules as through thoracotomy.

##### Strategy B

If aortic arch repair was indicated, it was performed as described previously through sternotomy. The ASO was performed in the basic manner, except few details:

- The atrial septal defect (ASD) was closed when the TV was normal or restricted when TV was small (z score ≤−3) to promote blood flow to the RV to allow growth potential. As the result of aortopulmonary discrepancy, coronary reimplantation was mostly achieved with the button technique, not to enlarge excessively the neoaortic root.
- This aortopulmonary discrepancy also needed specific re-establishment of the neo-pulmonary continuity with posterior sinuses reconstruction with oversized fresh autologous pericardial patch to enlarge the root.
- In case of anterior deviation of the infundibular septum, the right ventricular outflow tract (RVOT) was systematically cleared from any muscular band and sometimes undermining/thinning of the infundibular septum itself.
- PAB was a partially adjustable band made by a strip of polytetrafluoroethylene and tied with a series of 6/0 polypropylene, placed between the valve and the bifurcation, on the patch reconstruction itself.

Left atrial pressure was monitored to appreciate LV function and pulmonary venous return, and temporary atrial and ventricular pacing were placed.

#### Second stage

VSD(s) was/were closed through the TV, except for apical ones. In cases of double-outlet right ventricle, the patch was tied to the inferior border through the TV, and if access to the superior border was too difficult, it was tied through the neoaortic valve. In cases of TV straddling, the papillary muscles were repositioned to allow VSD closure. TV competence was tested and repair was performed when needed. PA trunk enlargement was performed in all patients at the site of PAB.

### Follow-Up and Analysis

Each patient had close follow-up with his/her cardiologist (every 2-3 months the first year; every 6-12 months afterwards). Timing for second surgery was discussed in multidisciplinary staff according to clinical and echocardiographic presentation. Unplanned reoperation after first stage could occur according to patient's clinical state and was defined as having to perform additional palliation before second stage, or to perform second stage before 5 kg of weight.

Results are expressed as frequencies and percentages for qualitative data. For continuous data, results are expressed as mean ± standard deviation for normal data and as median with its interquartile range (IQR) for non-normal data (first quartile to third quartile). Normality of variables were tested using the Shapiro-Wilk test. Comparisons between means of groups was tested using the Student *t* test for normal data and Wilcoxon-Mann-Whitney *U* test for non-normal data. The date of first occurrence of reoperation was recorded for time-to-event calculation. Cumulative incidences for the outcomes were estimated within the framework of competing risks, with death as a competing event; comparison was calculated with the Gray test.

## Results

### First Stage

**Figure 1:**
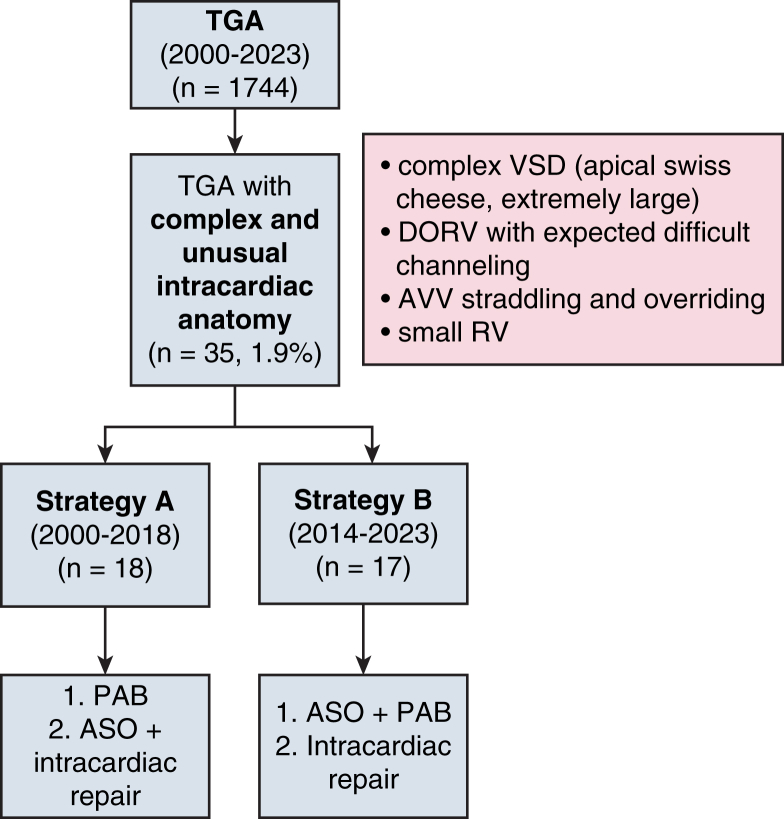
Patient population and summary of strategies. *TGA*, Transposition of the great arteries; *VSD*, ventricular septal defect; *DORV*, double-outlet right ventricle; *AVV*, atrioventricular valve; *RV*, right ventricle; *PAB*, pulmonary artery banding; *ASO*, arterial switch operation.

Thirty-five patients underwent first stage: 18 patients underwent strategy A (PAB) and 17 underwent strategy B (ASO + PAB) (see Figure 1 and Table 1; see Table E1 for a description of each patient’s anatomy). Although delayed sternal closure was more frequent when ASO was performed (*P* = .009), intensive care unit (ICU) stay (*P* = .69), mechanical ventilation (MV) time (*P* = 1), and inotrope duration (*P* = .95) were similar between strategies. When PAB was performed alone, inotropic support was more important (0.21; IQR, 0.20, 0.22 vs 0.13 μg/kg/min maximum epinephrine demand; IQR, 0.10, 0.15; *P* = .017) and oximetry was significantly lower at discharge (87.3% ± 5.2 vs 94.6% ± 4.3, *P* < .001).

**Table 1:** Patient perioperative data at the first stage

Strategy	A	B	*P* value
n (first surgery)	18	17	
Sex, n (%)			
Female	8 (44.4)	4 (23.5)	.34
Male	10 (55.6)	12 (76.5)	
Age, d, median [IQR]	15.0 [8.25, 26.75]	9.0 [6.0, 22.0]	.52
Weight, kg, mean (SD)	2.90 [2.49, 3.39]	3.29 [3.06, 3.71]	.16
Height, cm, median [IQR]	47.0 [45.25, 51.25]	48.5 [47.0, 49.62]	.29
Body surface area, m^2^, mean (SD)	0.20 (0.03)	0.22 (0.02)	.09
Balloon atrial septostomy, %	9 (50.0)	5 (29.4)	.37
Alprostadil requirement, %	5 (27.8)	7 (41.2)	.63
Coronary pattern, %∗			.41†
A	7 (38.9)	10 (58.8)	
B	1 (5.6)	1 (5.9)	
C	1 (5.6)	1 (5.9)	
D	2 (11.1)	0 (0.0)	
E	4 (22.2)	5 (29.4)	
Other	3 (16.7)	0 (0.0)	
Great arteries relationship, %			.27†
Anteroposterior	9 (50.0)	13 (76.5)	
Oblique	3 (16.7)	1 (5.9)	
Side-by-side	6 (33.3)	3 (17.6)	
Defect, %			.73†
TGA, VSD	12 (66.7)	10 (58.8)	
TGA, VSD, coarctation	6 (33.3)	7 (41.2)	
Aortic arch, %			.84†
Coarctation	3 (16.7)	4 (23.5)	
Hypoplastic	3 (16.7)	2 (11.8)	
Interrupted	0 (0.0)	1 (5.9)	
Normal	12 (66.7)	10 (58.8)	
VSD location, %			.2†
Inlet	1 (5.6)	3 (17.6)	
Outlet	10 (55.6)	4 (23.5)	
Outlet, trabeculated	4 (22.2)	8 (47.1)	
Trabeculated	3 (16.7)	2 (11.8)	
AVV straddling, %	4 (22.2)	3 (17.6)	1
DORV anatomy, %	10 (55.6)	6 (35.3)	.39
Small RV, %	1 (5.6)	3 (17.6)	.55
Additional procedure, %			.01†
Aortic arch repair	2 (11.1)	7 (41.2)	
Left and right AVV plasty, cleft closure	0 (0.0)	1 (5.9)	
Left PA plasty	0 (0.0)	1 (5.9)	
Aortic coarctation repair	4 (22.2)	0 (0.0)	
Right coronary ostium plasty	0 (0.0)	1 (5.9)	
VSD calibration (patch + hole)	0 (0.0)	1 (5.9)	
No	12 (66.7)	6 (35.3)	
CPB time, min, mean (SD)	90.3 (69.7)	179.9 (66.0)	.045
Aortic clamping time, min, median [IQR]	47.0 [43.5, 50.5]	97.0 [77.0, 124.0]	.024
Delayed sternal closure, %	2 (11.1)	10 (58.8)	.009
Duration of delayed sternal closure, d, mean (SD)	5.0 (0.0)	2.9 (1.4)	.065
ICU stay, d, median [IQR]	8.5 [4.75, 14.0]	7.0 [6.0, 8.5]	.69
Mechanical ventilation time, d, median [IQR]	3.0 [3.0, 9.0]	4.0 [2.5, 5.5]	1
Inotropes duration, d, median [IQR]	5.0 [3.0, 9.25]	5.0 [3.5, 6.0]	.95
Peak lactatemia, mmol/L, median [IQR]	3.6 [3.5, 4.7]	5.0 [2.9, 6.4]	.78
Peak epinephrine demand, μg/kg/min, median [IQR]	0.21 [0.20, 0.22]	0.13 [0.10, 0.15]	.017
Peak left atrial pressure, mm Hg, median [IQR]	12.0 [7.0, 12.25]	9.0 [9.0, 12.0]	.7
Trans-PAB velocity (before discharge), m/s, median [IQR]	4.0 [3.9, 4.0]	3.0 [2.75, 3.5]	.001
Spo_2_ (before discharge), %, mean (SD)	87.3 (5.2)	94.6 (4.3)	<.001

*IQR*, Interquartile range; *SD*, standard deviation; *TGA*, transposition of the great arteries; *VSD*, ventricular septal defect; *AVV*, atrioventricular valve; *DORV*, double-outlet right ventricle; *RV*, right ventricle; *PA*, pulmonary artery; *CPB*, cardiopulmonary bypass; *ICU*, intensive care unit; *PAB*, pulmonary artery banding; *Sp**o*_*2*_, saturation of peripheral oxygen.

∗ Yacoub and Radley-Smith coronary arteries pattern classification.

† Fischer's exact test.

**Figure 2:**
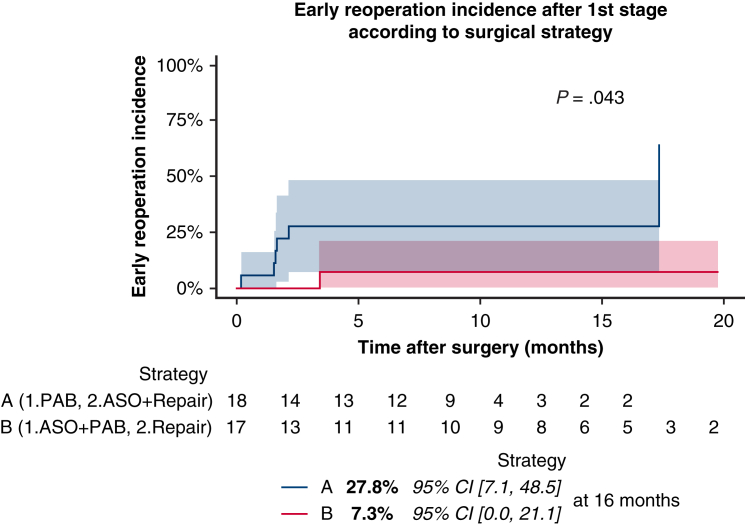
Cumulative incidence of unplanned reoperation after first stage, according to surgical strategy. *Shaded areas* represent 95% confidence interval. *PAB*, Pulmonary artery banding; *ASO*, arterial switch operation.

Unplanned reoperation incidence occurred more with strategy A (n = 6, 27.8%; 95% CI, 7.1, 48.5) than B (n = 1, 7.3%; 95% CI, 0.0, 21.1; *P* = .043) at 16 months after first stage (Figure 2). From strategy A, 1 patient (B′) underwent additional palliation (ASD enlargement and bicavopulmonary shunt for deep cyanosis with weight stagnation 18 months after surgery) and 5 (A′, C′, F′, H′, J’) early second stage (with less than 5 kg, for deep cyanosis (n = 3) and PAB migration (n = 2; one distally and one proximally threatening the neoaortic valve). From strategy B, 1 patient (F) underwent additional palliation 3 months postoperatively (mitral valve abnormal chordae section, PAB and VSD enlargement for double obstruction).

### Second Stage

Twenty-eight patients underwent second stage: 18 strategy A (ASO + intracardiac repair) and 10 strategy B (intracardiac repair) (see Table 2); 4 patients from strategy B were retransferred to their native country after the first stage, 1 patient died from cardiac causes and 2 from noncardiac causes (see to follow).

**Table 2:** Patient perioperative data at the second stage

Strategy	A	B	*P* value
n (second surgery), %	18	10	
Trans-PAB velocity before second surgery, m/s, median [IQR]	4.0 [4.0, 4.75]	4.6 [4.0, 5.0]	.54
Spo_2_, (before second surgery), %, mean (SD)	75.0 [70.5, 76.5]	87.0 [85.25, 89.0]	.005
Age, mo, median [IQR]	8.40 [3.22, 10.12]	15.50 [12.38, 18.40]	.007
Time between stages, mo, median [IQR]	8.06 [2.93, 9.38]	15.35 [12.10, 18.17]	.006
Weight, kg, median [IQR]	6.35 [4.44, 7.72]	9.90 [8.55, 10.73]	.005
Height, cm, median [IQR]	65.8 [64.0, 72.0]	76.0 [74.25, 81.0]	.025
Body surface area, m^2^, mean (SD)	0.35 (0.12)	0.45 (0.09)	.022
Second stage procedure, %			<.001
ASO, VSD closure, PA trunk enlargement	17 (94.4)	0 (0.0)	
Bex-Nikaidoh, VSD closure, PA trunk enlargement	1 (5.6)	0 (0.0)	
VSD closure, PA trunk enlargement	0 (0.0)	10 (100.0)	
Additional procedure, %			.12
Left and right AVV plasty	0 (0.0)	1 (10.0)	
BDGS	0 (0.0)	1 (10.0)	
Restricted ASD, %	2 (11.1)	5 (50.0)	.069
CPB time, min, mean (SD)	257.38 (69.98)	159.09 (29.72)	<.001
Aortic clamping time, min, median [IQR]	148.29 (26.90)	86.27 (18.26)	<.001
Delayed sternal closure, %	8 (44.4)	0 (0.0)	.071
Duration of delayed sternal closure, d, median [IQR]	3.0 [2.0,3.75]		
ICU stay, d, median [IQR]	7.50 [5.75, 9.00]	2.00 [2.00, 2.75]	.002
Mechanical ventilation time, d, median [IQR]	2.50 [1.00, 6.00]	1.00 [0.00, 1.00]	.006
Inotropes duration, d, median [IQR]	4.00 [2.00, 6.00]	1.00 [0.25, 1.00]	.002
Peak lactate, mmol/L, mean (SD)	3.80 (0.81)	3.00 (1.52)	.210
Peak epinephrine demand, μg/kg/min, median [IQR]	0.15 [0.12, 0.20]	0.10 [0.10, 0.13]	.177
Follow-up time, y, median [IQR]	8.13 [1.38, 13.77]	1.27 [0.94, 1.62]	.02

*PAB*, Pulmonary artery banding; *IQR*, interquartile range; *Sp**o*_*2*_, saturation of peripheral oxygen; *SD*, standard deviation; *ASO*, arterial switch operation; *VSD*, ventricular septal defect; *PA*, pulmonary artery; *AVV*, atrioventricular valve; *BDGS*, bidirectional Glenn shunt; *CPB*, cardiopulmonary bypass; *ICU*, intensive care unit.

Before surgery, oximetry was lower with strategy A (saturation of peripheral oxygen = 75.0%; IQR, 70.5, 76.5) than B (saturation of peripheral oxygen = 87.0%; IQR, 85.25, 89.0; *P* = .005). Patients who received strategy A underwent second-stage repair earlier (time interval: 8.1 months; IQR, 2.9, 9.4 vs 15.3 months; IQR, 12.1, 18.2; *P* = .006) at lower weights (6.3 kg; IQR, 4.4, 7.7 vs 9.9 kg; IQR, 8.5, 10.7, *P* = .005). One patient from strategy A (I′) underwent a Bex-Nikaidoh procedure for pulmonary root infective aneurysm with partial destruction of the valve, discovered intraoperatively.

After surgery, patients who received strategy A had longer stays in the ICU (7.5 days; IQR, 5.75, 9.0) vs 2.0 days; IQR, 2.0, 2.75; *P* = .002), MV time (2.5 days; IQR, 1.0, 6.0 vs 1.0 day; IQR, 0.0, 1.0), *P* = .006), and inotrope duration (4.0; IQR, 2.0, 6.0 vs 1.0 day; IQR, 0.25, 1.00; *P* = .002).

At 30 months after second stage, 3 unplanned reinterventions occurred, all in patients who received strategy A (18.6%; 95% CI, 0.0-37.9 vs 0%, *P* = .23). One patient (F′) underwent RVOT enlargement for subpulmonary stenosis at 20 months, one (J′) underwent aortic arch enlargement and RV-PA conduit placement for persistent coarctation and hypoplastic pulmonary annulus at 1 month, and one (M′) underwent PA trunk enlargement and extracorporeal life support weaning after biventricular dysfunction with major hypertrophy, at 8 days.

### Survival

Deaths from cardiac cause occurred in one patient in each strategy: one (I′) from pulmonary root endocarditis at 7 months after second stage (strategy A) and one (B) from RV failure secondary to a right coronary ostium atresia discovered intraoperatively at 7 days after first stage (strategy B). Deaths from noncardiac causes occurred in 2 patients from strategy B after first stage: one (K) from pyocyanic infection and one (F) from severe lung injury.

### Complications

Ventilatory complications (including pneumonia, need for high-frequency ventilation, and causes preventing mechanical ventilation weaning: pulmonary overflow, breaks, deep cyanosis, PAB displacement, diaphragmatic paralysis) occurred more in strategy A (55.6% [n = 10] vs 17.6% [n = 3], *P* = .049) (see Table 3 for overall complications and Table E2 for complications after each stage).

**Table 3:** Complications after both stage surgeries

Strategy	A	B	*P* value
Bleeding re-exploration, %	2 (11.1)	2 (11.8)	1
LCOS, %	3 (16.7)	5 (29.4)	.62
Arrythmia, %	1 (5.6)	3 (17.6)	.55
Ventilatory complication, %	10 (55.6)	3 (17.6)	.049
Pneumonia, %	7 (38.9)	3 (17.6)	.31
Stroke, %	0 (0.0)	1 (5.9)	.98
ECLS, %	1 (5.6)	2 (11.8)	.96
Endocarditis, %	1 (5.6)	0 (0.0)	1
RVOT/PA trunk stenosis, %	5 (27.8)	0 (0.0)	.22
Atrioventricular block, %	0 (0.0)	1 (5.9)	.98
Death from cardiac cause, %	1 (5.6)	1 (5.9)	1

*LCOS*, Low cardiac output state; *ECLS*, extracorporeal life support; *RVOT*, right ventricle outflow tract; *PA*, pulmonary artery.

## Discussion

This study presents a novel staged strategy for patients with TGA associated with complex intracardiac anatomy which, in our institution, concerns a small proportion of all TGA (1.9%). It aims to avoid the risks inherited from the “classical” staged repair (ie, 1/PAB and 2/ASO with intracardiac repair) such as damage of the neoaortic valve, hypertrophy leading to subvalvular stenosis, early mortality^10^ and late neo-aortic root dilation and regurgitation.^17^ It also aims to avoid risks related to a 1-stage-repair with too early VSD closure such as atrioventricular block, residual VSD, and ventricular dysfunction. This strategy may also act as a palliation for univentricular hearts with ventriculoarterial discordance (such as double inlet left ventricle or tricuspid atresia, with or without subaortic stenosis), as an alternative of isolated PAB of Norwood type procedures; it presents the theoretical advantages of aligning the single systemic ventricle with the unobstructed posterior semilunar valve.^19^ Strategy B is also preferrable in case of “1.5 ventricle” repair. As seen in our cohort, when the TV was small at first stage (z score at −3) but not atretic, a chance could be given to enhance RV growth until second-stage surgery, where it can be decided between 2 and 1.5 ventricle repair (with bicavopulmonary shunt). A restricted ASD should be created in this case.

PAB with a Gore-Tex band that is passed around the reconstructed PA trunk, following the well-established rule of Trusler and Mustard,^18^ is a simple and quick procedure that doesn't require longer periods of assistance than standard ASO and is placed under direct view. Other methods^20^^,^^21^ such as restrictive Gore-Tex tube require longer cardiopulmonary bypass times, have 2 parameters impacting the flow (diameter and length), and lack the possibility of adjusting the PAB after its completion. Though PAB were all made adjustable, no patient in our cohort required percutaneous PAB dilatation between first and second stages; patients were instead referred for second stage, especially if they have reached sufficient growth, allowing for a low risk intracardiac repair. However, we think that making PAB systematically adjustable could still be useful as it theoretically allows longer time interval until second stage.

In our study, time interval between stages was different according to the strategy. The second stage was systematically earlier in strategy A than B, because patients had deeper cyanosis, given the not ideal blood flow streaming in this anatomy. As a result, sufficient weight was not reached by most of the patients because they couldn't wait any longer for next surgery, and greater risk was taken when performing too early intracardiac repair.

Furthermore, early anatomic repair appears also interesting for patients from developing countries with uncertain follow-up, as we experienced with strategy B. After first stage, in addition to the fact that they could wait longer until second stage, best management of their intracardiac repair was easier to plan (ie, 1 patient from a developing country, who underwent at 1 year of age ASO + PAB [strategy B] with pulmonary biopsy, had his VSD “restricted” at first stage that would be closed percutaneously later, along with PAB dilation, according to biopsy results).

Three deaths occurred in strategy B. Given the complex anatomy of most of these patients, we believed that 2 of these deaths were not directly imputable to the strategy itself: one had right coronary ostium atresia and one had pyocyanic infection. However, 1 death could be attributable to the strategy, as the unplanned reintervention for double obstruction included VSD and PAB enlargement, which led to severe lung injury. In group A, the only death that occurred was directly imputable to PAB as the primary cause was an infectious aneurysm of the pulmonary root, just below the PAB.

Reasons for reoperation after second stage in strategy A all included RVOT/pulmonary trunk obstruction in 3 patients and persistent coarctation in 1 patient. The latter appears avoidable if a full aortic arch enlargement is performed at first stage through sternotomy. Right outflow obstruction may be a result of the strategy A itself, but no difference was highlighted with our small cohorts.

We thus believe that optimal surgical option for these patients is a 2-stage strategy with ASO performed early. In addition, placing the PAB around the anterior vessel (after ASO) allows good exposure to adjust it properly.

### Limitations

This study is a retrospective monocentric study in a high-volume center. Two different eras are compared, and it is difficult to say whether the management of patients may have slightly changed over this period. Follow-up time is shorter in strategy B (more recent). A proportion of the patients was lost to follow-up, most of the time after returning to their country of origin, after first stage of strategy B. The study is based on a small cohort of patients, and differences between our 2 strategies can be difficult to highlight because it concerns a rare heart defect. However, as limited data exists about patients with TGA associated with complex intracardiac anatomy, this study might be a good start for larger cohorts.

## Conclusions

The staged repair strategy for TGA associated with complex intracardiac anatomy, consisting of performing ASO + PAB as first surgery (over PAB alone) allows intracardiac repair after sufficient growth of the patient, prevents ventilatory complications, and decreases ICU stay, MV time, and inotrope duration after intracardiac repair. Further data with larger cohorts and longer follow-up will be necessary to assess superiority of this strategy, but this study is a significant start for a better management of these complex patients.

## Conflict of Interest Statement

The authors reported no conflicts of interest.

The *Journal* policy requires editors and reviewers to disclose conflicts of interest and to decline handling or reviewing manuscripts for which they may have a conflict of interest. The editors and reviewers of this article have no conflicts of interest.

## Figures and Tables

**Table E1:** Analysis of individual patient defects and repair strategy

Patient ID	Age, d	Body surface, m^2^	Coronary pattern∗	Great arteries relationship	Intracardiac anatomy	Associated intracardiac defects	Aortic arch	Procedure (first stage)
Strategy A								
A'	13	0.18	E	SS	dTGA, outlet VSD, muscular VSDs		Normal	PAB
B'	43	0.21	A	AP	dTGA, outlet VSD, muscular VSDs		Normal	PAB
C'	28		B	O	dTGA, DORV		Normal	PAB
D'	8		E	SS	dTGA, muscular VSDs		Normal	PAB
E'	48	0.21	A	AP	dTGA, perimembranous extended to inlet VSD, muscular VSDs	Small RV	Normal	PAB
F'	19	0.2	E	AP	dTGA, muscular VSDs		Coarctation	Aortic coarctation repair, PAB
G'	21	0.23	A	AP	dTGA, muscular VSDs	TV straddling	Normal	PAB
H'	23	0.22	A	AP	dTGA, DORV, muscular VSDs		Coarctation	Aortic coarctation repair, PAB
I'	17	0.19	LAD from RCA	AP	dTGA, DORV, large outlet extended to inlet VSD		Normal	PAB
J'	6	0.15	E	SS	dTGA, DORV		Coarctation	Aortic coarctation repair, PAB
K'	13	0.17	D	O	dTGA, DORV	MV straddling	Normal	PAB
L'	11	0.19	RCA from left ant. sinus; LCA from left post.	O	dTGA, DORV, large outlet extended to inlet VSD, muscular VSDs	Superposed ventricles	Normal	PAB
M'	9	0.19	A	AP	dTGA, outlet VSD, muscular VSDs		Hypoplastic	Aortic coarctation repair, PAB
N'	29	0.24	D	AP	dTGA, DORV	TV straddling	Normal	PAB
O'	7	0.19	A	SS	dTGA, DORV, muscular VSDs	MV straddling	Normal	PAB
P'	28	0.25	C	AP	dTGA, DORV		Normal	PAB
Q'	3	0.16	A	SS	dTGA, DORV		Hypoplastic	Aortic arch repair, PAB
R'	6	0.17	Unique RCA	SS	dTGA, DORV		Hypoplastic	Aortic arch repair, PAB
Strategy B								
A	9	0.2	A	AP	dTGA, large outlet VSD, muscular VSDs		Hypoplastic	Aortic arch repair, ASO, PAB
B	22	0.18	E	SS	dTGA, DORV, muscular VSDs	Right coronary ostium atresia	Normal	ASO, right coronary ostium plasty, PAB
C	6	0.19	A	AP	dTGA, outlet VSD, muscular VSDs		Normal	ASO, PAB
D	22	0.25	A	AP	dTGA, large inlet VSD	TV overriding, small RV	Normal	ASO, PAB
E	6	0.21	A	AP	dTGA, large inlet VSD	MV cleft, TV straddling, small RV	Coarctation	Aortic arch repair, ASO, PAB
F	30	0.21	C	AP	dTGA, DORV, muscular VSDs	Subaortic stenosis, MV cleft	Normal	ASO, PAB
G	154	0.3	A	AP	dTGA, DORV, large outlet extended to inlet VSD	MV straddling, hypoplastic left PA	Normal	ASO, Left PA plasty, PAB
H	334	0.31	E	AP	dTGA, outlet VSD		Normal	VSD calibration (patch + hole), ASO, PAB
I	5	0.23	A	AP	dTGA, muscular VSDs		Normal	ASO, PAB
J	4	0.22	A	AP	dTGA, outlet VSD, muscular VSDs		Normal	ASO, PAB
K	11	0.24	B	AP	dTGA, DORV, muscular VSDs		Hypoplastic	Aortic arch repair, ASO, PAB
L	93	0.27	A	AP	dTGA, inlet VSD	MV cleft	Normal	Left and right AVV plasty, MV cleft closure, ASO, PAB
M	6	0.2	A	AP	dTGA, outlet VSD, muscular VSDs		Coarctation	Aortic arch repair, ASO, PAB
N	20	0.21	E	O	dTGA, DORV, large outlet extended to inlet VSD	MV cleft	Interrupted	Aortic arch repair, ASO, PAB
O	5	0.21	E	SS	dTGA, DORV, muscular VSDs	MV cleft	Coarctation	Aortic arch repair, ASO, PAB
P	8	0.21	E	SS	dTGA, outlet VSD	Small RV	Coarctation	Aortic arch repair, ASO, PAB
Q	7	0.20	A	AP	dTGA, muscular VSDs		Normal	ASO, PAB

*SS*, Side-by-side; *dTGA*, transposition of the great arteries; *VSD*, ventricular septal defect; *PAB*, pulmonary artery banding; *AP*, anteroposterior; *O*, oblique; *DORV*, double-outlet right ventricle; *RV*, right ventricle; *LAD*, left anterior descending; *RCA*, right coronary artery; *MV*, mitral valve; *TV*, tricuspid valve; *ASO*, arterial switch operation; *PA*, pulmonary artery; *AVV*, atrioventricular valve.

∗ Yacoub and Radley-Smith coronary arteries pattern classification.

**Table E2:** Complications after each stage surgeries, separately

Strategy	A	B	*P* value
After first stage			
Bleeding re-exploration, %	1 (5.6)	2 (11. 8)	.53
LCOS, %	2 (11.2)	5 (29.4)	.19
Arrythmia, %	1 (5.6)	3 (17.6)	.27
Ventilatory complication, %	9 (50.0)	2 (11.8)	.014
Pneumonia, %	3 (16.8)	3 (17.6)	.94
Stroke, %	0 (0.0)	1 (5.9)	.31
ECLS, %	0	2 (11.8)	.14
Death from cardiac cause, %	0	1 (5.9)	.31
After second stage			
Bleeding re-exploration, %	1 (5.6)	0	.47
LCOS, %	1 (5.6)	0	.47
Ventilatory complication, %	5 (27.8)	1 (10.0)	.29
Pneumonia, %	5 (27.8)	0	.07
ECLS, %	1 (5.6)	0	.47
Endocarditis, %	1 (5.6)	0 (0.0)	.47
RVOT/PA trunk stenosis, %	5 (27.8)	0 (0.0)	.07
Atrioventricular block, %	0 (0.0)	1 (10.0)	.19
Death from cardiac cause, %	1 (5.6)	0	.47

*LCOS*, Low cardiac output state; *ECLS*, extracorporeal life support; *RVOT*, right ventricle outflow tract; *PA*, pulmonary artery.
